# Comparing the Networks that Power Bacterial Chemotaxis

**DOI:** 10.1371/journal.pbio.0020066

**Published:** 2004-02-17

When we think of foraging for food, we usually imagine animals wandering in the woods, poking behind bushes and trees, trying to find something tasty. Amazingly, even single-cell bacteria display a simplified version of this behavior. Many species of bacteria can respond to chemical or nutritional cues (chemoattractants) in the environment by moving toward locations with more favorable conditions, a process known as chemotaxis. The bacteria adjust their movements by rotating threadlike projections called flagella either clockwise or counterclockwise; these adjustments are made by a network of proteins in response to chemoattractants. Chemotaxis has been identified in many bacterial species, but two of the best-studied examples are in Escherichia coli and Bacillus subtilis. Computer modeling of chemotaxis in these species now reveals some important differences in the network architecture that controls this complex behavior.

Most of the proteins involved in chemotaxis in E. coli and B. subtilis have been identified and well studied, but much remains to be learned about this biological process. As scientists have begun to understand how the proteins work together, they're discovering a network of interactions that operates a bit like an electronic circuit. Researchers have found that using the circuit as a model for protein networks has helped them to understand how complex system properties arise from seemingly simple interactions between proteins. These properties can be explored with the aid of computer simulations, whereby researchers can rapidly test a given system under many different situations and can tweak the properties of the proteins and their connections.

The team, led by Adam Arkin of the University of California at Berkeley, has compared the system level properties of chemotaxis in the two bacterial species E. coli and B. subtilis. Not surprisingly, the proteins involved in the signaling pathway are conserved—that is, they have changed very little since they first evolved—even though these species are evolutionarily very distant. In many cases, a gene from one species can even substitute for the ortholog (a conserved gene that retains the same function even though two species have diverged) in the other. Despite these similarities, however, disrupting the function of orthologous genes in these two species often has different, even opposite, effects. This is surprising, especially given that the chemotactic behaviors of E. coli and B. subtilis are almost identical. In order to understand this puzzling observation, the researchers constructed a network model of the chemotaxis system in B. subtilis and used simulations to understand how the network properties differ from those of existing models of E. coli chemotaxis.

The group found that despite the similarities in proteins and the nearly identical behavior between the two species, the mechanisms underlying the behavior are quite distinctive. When comparing the system properties of these two bacterial systems, the researchers also made an unusual observation. Though the two “circuits” have different wiring, the system properties underlying the behavior, called the control strategy, are very similar. The two species of bacteria therefore achieve the same chemotaxis behavior by using similar proteins, but in different ways.

Arkin and colleagues draw two important conclusions from these results. First, these two systems have conserved proteins, but the proteins are wired together differently. This means that wiring of signaling networks cannot be inferred simply by identifying the conserved proteins in the network. Second, in these systems, conserved proteins use different mechanisms to accomplish the same overall control strategy. This raises the question of how such systems evolve. The authors suggest that the control strategy itself may be an evolutionarily conserved property. These conclusions will be important to keep in mind as researchers examine these systems in more detail and begin to examine more complex systems as well.

**Figure:**
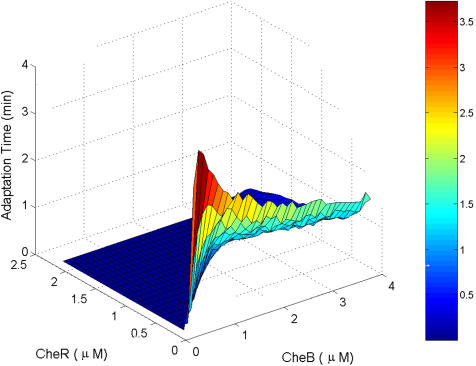
Modeling chemotaxis

